# Effects of Enzymatic Hydrolysis on Physicochemical Properties and Solubility and Bitterness of Milk Protein Hydrolysates

**DOI:** 10.3390/foods10102462

**Published:** 2021-10-15

**Authors:** Qiang Cui, Yuxue Sun, Zengjia Zhou, Jianjun Cheng, Mingruo Guo

**Affiliations:** 1 Key Laboratory of Dairy Science, Northeast Agricultural University, Harbin 150030, China; cuiqiangwx@163.com (Q.C.); sunffyy@163.com (Y.S.); m15255825668@163.com (Z.Z.); jjcheng@neau.edu.cn (J.C.); 2 Department of Nutrition and Food Sciences, College of Agriculture and Life Sciences, University of Vermont, Burlington, VT 05405, USA

**Keywords:** milk protein concentrate, solubility, bitterness, physicochemical properties

## Abstract

Milk protein concentrate (MPC) is a high-protein dairy product. It is underutilized due to its poor solubility compared with other milk protein products. This study aimed to investigate the effect of enzymatic hydrolysis on the physicochemical properties and solubility of MPC. Results showed that Alcalase hydrolysates possessed a higher degree of hydrolysis (DH) than Protamex and Flavourzyme hydrolysates. Similar results could be obtained using sodium dodecyl sulfate–polyacrylamide gel electrophoresis (SDS-PAGE). The molecular weight of the hydrolysate of Alcalase was less than 10 kDa. Changes to the molecular weight thereby led to a modification in the fluorescence intensity, Fourier-transform infrared spectrometry, and ultraviolet absorption. The solubility of all hydrolysates was significantly increased (*p* < 0.05). Correlation analysis showed a positive correlation between solubility, DH, and bitterness; the correlation coefficients were 0.81 for DH and 0.61 for bitterness. Electronic tongue analysis showed that the bitterness of Alcalase hydrolysates was the highest, while the values for Protamex hydrolysates were the lowest.

## 1. Introduction

Milk protein concentrate (MPC) is an ideal ingredient for high-protein foods. It has excellent functional properties, is rich in essential amino acids, and has high digestibility, thus making it an ideal choice for various nutritional products [1]. MPC is different from whole milk powder (WMP) and skim milk powder (SMP). It is a product with minimum levels of lactose prepared from skim milk by physical separation techniques, such as membrane filtration [2,3]. The protein content is higher, generally 42–85% [4]. The main proteins in MPC are whey protein and casein, and the ratio of casein to whey protein (80:20) is similar to that in skim milk [5]. At present, MPC is widely used in high-protein products, such as nutrition bars, coffee creamers, beverages, processed cheese, and Greek-style yogurt [6]. However, solubility is affected by a combination of temperature, relatively high relative humidity, and water activity. McKenna [7] reported the formation of insoluble substances in the MPC powder. It was found that the insoluble matter was formed by casein micelles, with a particle size of up to 100 μm, and these micelles appear to aggregate through some form of protein-protein interaction. Havea [8] observed that the insoluble matter in the MPC powder increased with the storage time at high temperatures. This substance is mainly composed of α-casein and β-casein. Anema, Pinder, Hunter, and Hemar [9] found that the insolubility in MPC might be caused by the protein cross-linking on the surface. Additional evidence showed that protein dissociates from casein micelles (especially κ-casein) and aggregates with β-lactoglobulin, forming a “skin” on the surface of the powder particles and causing insolubility [10]. However, other mechanisms cannot be excluded, such as cross-linking of the proteins by hydrophobic and/or hydrogen bonding [10].

The solubility of a protein is the result of a balance between hydrophobic and ionic interactions. Hydrophobic interaction facilitates the interaction between the proteins, and ionic interaction promotes the interaction between protein and water [11]. In general, protein-protein interactions are more unfavorable for dissolution than protein-water interactions. A decrease in solubility will have a higher impact on other functional properties (gelation, foaming, emulsification, and surface activity) of the protein [12], thus limiting their use in yogurt, nutritional beverages, gel food, and cheese. Le, Bhandari, and Deeth [13] found that the Maillard reaction between lactose and protein may be one of the reasons for a decrease in the solubility of MPC powder. Ye [14] studied the relationship between calcium content in MPC and casein aggregation and found that the total protein concentration on the surface of low-calcium MPC emulsions was lower in comparison. The protein composition of the droplet surface had changed, and the aggregation state of casein influenced the emulsifying ability of MPC. Sun et al. [15] found that longer ultrasound pretreatment time induced more significant changes in the MPC function, such as gelation, solubility, and emulsification. These changes may be due to a destruction of the aggregation state of the MPC protein by sonication. Since caseins in MPC are easy to digest, it is easier to be hydrolyzed by the enzymes than the dense spherical structure of the whey protein [16]. Therefore, enzyme treatment decreases the aggregation state and improves the functional properties of MPC. A number of studies reported an improvement in protein solubility by enzymatic hydrolysis [17,18,19]. The functional properties of MPC and its hydrolysates are of extreme importance. Flavourzyme, Alcalase, and Promatex are widely used in the production of hydrolyzed products. Flavourzyme is produced from Aspergillus oryzae strains and is used for the hydrolysis of protein under neutral or slightly acid conditions. It is an aminopeptidase, i.e., a mixture of peptidase, endopeptidase, and exopeptidase. Flavourzyme hydrolyzed products formed by being mixed with bitter peptidase are diverse and usually small peptides and amino acids. Alcalase has a high specificity for aromatic (Phe, Trp, and Tyr), acidic (Glu), sulfur-containing (Met), aliphatic (Leu and Ala), hydroxyl (Ser), and basic (Lys) residues, which preferentially hydrolyses peptide bonds containing aromatic amino acid residues. Promatex is suggested for animal protein extraction as it contains vastly broad-spectrum endo-proteases, allowing an extensive hydrolysis of proteins. Severin and Xia [20] studied Alcalase and Protamex hydrolysates of whey protein concentrate (WPC80) at different degrees of hydrolysis (DH). They reported improved protein solubility due to the formation of small particles. However, it is well known that protein hydrolysis may result in a bitter taste, mainly due to the presence of bitter peptides. Therefore, the choice of enzymes is a highly crucial factor.

Understanding the hydrolysis of the two main proteins of casein and whey protein in MPC is necessary to improve the applicability of dairy products. However, the literature on the enzyme hydrolysis of MPC is limited [21], especially the knowledge of the structure and functional properties of MPC. Therefore, this study aimed to investigate: (1) the effects of different hydrolysis times (30, 60, 90, 120, or 180 min) on the structural properties by UV spectra, intrinsic fluorescence spectroscopy, surface hydrophobicity, and FTIR test; and (2) three enzymes—Alcalase (endopeptidase), Flavourzyme, and Protamex—containing both endo- and exo-peptidase on the physicochemical properties and the relationship between the solubility and bitterness of MPC.

## 2. Materials and Methods

### 2.1. Materials

Milk protein concentrate-85 was purchased from Ingredia Pte Ltd. (Midview, Singapore; manufacture date: 27 November 2019). The conditions of storage were 25 ± 3 °C, and the MPC was stored for 6 months and then studied. Flavourzyme (500 LAPU g^−1^, both with endo- and exo-peptidase), Protamex (1.5 AU g^−1^, both with endo- and exo-peptidase), and Alcalase (2.4 AU g^−1^, endopeptidase) were purchased from Novo Co. (NovoNordisk, Bagsvaerd, Denmark). Other chemicals and reagents used in this study were analytical grades.

### 2.2. Enzyme Hydrolysates

MPC was mixed into ultrapure water (MilliQ system; Millipore, Billerica, MA, USA) to obtain a 5% solution (*w*/*v*). We used 1 M HCl and 1 M NaOH to maintain the pH of the total volume of the MPC solutions (300 mL). The Alcalase (enzyme/substrate weight ratio, 1:50) was hydrolyzed at 55 °C and pH 8.5. Protamex (enzyme/substrate weight ratio, 1:50) was hydrolyzed at 50 °C and pH 6.5, and Flavourzyme (enzyme/substrate weight ratio, 1:50) at 50 °C and pH 7. The pH of the total volume of the MPC solutions was maintained at the optimum value for each enzyme during the hydrolysis process. Based on preliminary hydrolysis work, the aliquots hydrolysates (50 mL) were collected at time intervals of 30, 60, 90, 120, 150, and 180 min and the aliquots were heated in a water bath (90 °C) for 15 min to deactivate enzymes. Its pH was then adjusted to 7.0 using HCl or NaOH. The samples were centrifuged at 8000× *g* for 15 min and the supernatant was collected and freeze-dried. All experiments were repeated three times.

### 2.3. Degree of Hydrolysis (DH)

The DH of the samples was determined according to the method of Zheng et al. [19]: briefly, at room temperature (22–28 °C), mixed with o-phthaldialdehyde (OPA) reagent (3 mL) and MPC hydrolysate (400 μL), and then incubated accurately for 2 min. At 340 nm, the absorbance of the mixed solution was measured with a UV spectrophotometer.

### 2.4. SDS-PAGE

Electrophoresis of MPC hydrolysate was determined according to the method reported by Laemmli [22]. In particular, the concentrated gel (5%) and separated gel (12%) were prepared for SDS-PAGE electrophoresis experiments and the sample was diluted to 5 mg/mL (10 μL), mixed with SDS loading buffer, and boiled for 5 min. The voltage was set as 80 V when electrophoresis was initiated, and as 120 V when the strip entered the separation gel. At the end of electrophoresis, we stained the gel with Coomassie Brilliant Blue Rapid Staining Solution (Solarbio Co., Ltd., Beijing, China) for 1 h. De-staining: with an ultrapure water for decolorization treatment; each treatment was 30 min; a total of five treatments.

### 2.5. Ultraviolet (UV) Spectra

The MPC hydrolysates were dissolved in 0.01 mol/L, pH 7.0 phosphate-buffered saline (PBS), and the protein content was diluted to 0.1 mg/mL. Then, following the method of Avramenko, Low, and Nickerson [23], we set the scanning wavelength range of 250–480 nm to UV scanning.

### 2.6. Intrinsic Fluorescence Spectroscopy

MPC hydrolysates were dissolved in 0.01 mol/L, pH 7.0 PBS and the protein content was diluted to 0.2 mg/mL. We then set the excitation wavelength to 290 nm and the emission wavelength was from 300 to 450 nm. The fluorescence spectrophotometer (F-4500, Hitachi, Tokyo, Japan) performed the scanning [24].

### 2.7. Surface Hydrophobicity (H_0_) Measurements

H_0_ of MPC hydrolysate was determined according to the method reported by Hu, Cheung, Pan, and Li [25]. Briefly, MPC hydrolysate protein content was adjusted to 0.025, 0.050, 0.100, and 0.200 mg/mL, then mixed with 1-aniline naphthalene-8-sulfonic acid solution (ANS, 20 μL) and kept in the dark for 15 min. The emission wavelength was 470 nm and the excitation wavelength was 390 nm. The surface hydrophobicity of the protein was expressed as the initial slope of relative fluorescence intensity (RFI) relative protein concentration (calculated by linear regression analysis).

### 2.8. Fourier Infrared Spectrum (FTIR)

FTIR of MPC hydrolysate samples was determined by Silva, Zisu, and Chandrapala [26]. We mixed the MPC hydrolysate sample (1 mg) with potassium bromide, then set the measurement range at 4000–400 cm^−1^. FTIR-8400 S spectrometer was used for FTIR measurement.

### 2.9. Static Laser Light Scattering for Particle Size

MPC hydrolysates were dissolved in 0.2 mol/L, pH 7.0 PBS and the protein content was diluted to 0.1 mg/mL. Then untreated MPC was measured by Master Sizer 3000 E laser particle size analyzer (Malvern Instruments Ltd., Malvern, UK), and the MPC hydrolysates samples were measured by NANO ZS 90 laser particle size analyzer (Malvern Instruments Ltd., Malvern, UK) [27].

### 2.10. Electronic Tongue Measurements

The taste changes of samples were determined according to the method reported by Liu, Zhu, Peng, Guo, and Zhou [28]. Briefly, MPC hydrolysate protein content was adjusted to 0.02 g/mL. Electronic Tongue TS-5000 Z (Insent Inc., Tokyo, Japan) was used for taste measurement. The reference solution was prepared by dissolving tartaric acid and potassium chloride in ultrapure water. Each sample was measured four times with the sensors of bitterness aftertaste (aftertaste-B), astringency, astringent aftertaste (aftertaste-A), and bitterness.

### 2.11. Soluble Protein Determination

The untreated MPC and MPC hydrolysates were magnetically stirred for 30 min to fully disperse in ultrapure water (1%, *w*/*v*). Briefly, the content of soluble protein in the supernatant was determined with the Biuret method. The calculation formulations of solubility were as follows [29]:

Solubility = Soluble protein content in hydrolysate × Volume of hydrolysate/Total protein × 100(1)

### 2.12. Statistical Analysis

Experiments were carried out three times and the mean and standard deviation were calculated from this data. Differences between the values at different incubation times for each enzyme were analyzed using ANOVA, and *p* < 0.05 was identified as being significantly different. All figures were plotted using Origin 2020 (OriginLab Corporation, Northampton, MA, USA).

## 3. Results

### 3.1. Degree of Hydrolysis and Analysis of Soluble Protein

The DH of MPC hydrolysates obtained from Alcalase, Protamex, and Flavourzyme digestion are presented in Table 1. The DH showed increasing trends in different hydrolysis times and exhibited a sharp increase in the first 30 min. Alcalase showed a much faster rate of hydrolysis than Protamex and Flavorzyme, which was consistent with the results of Al-Ruwaih, Ahmed, Mulla, and Arfat [30]. After 120 min, the DH displayed a relative plateau-like pattern. The change process of DH in this study was similar to those in previous studies [17,31,32,33]. The DH (180 min) of 15.72% was observed when hydrolyzing MPC with Alcalase, while the DH (180 min) of Protamex and Flavourzyme were 13.62% and 11.87%, respectively. The aforementioned proteases behaved differently even at the optimum temperature and pH, further indicating that the difference in hydrolysates was determined by the specific reaction site of the protease.

MPC powders gradually lose protein solubility upon storage [34]. Table 1 shows the solubility of MPC. As the insoluble material was formed, more of the soluble protein turned insoluble and was lost to the sediment during centrifugation. Following the hydrolysis of MPC, the solubility of the hydrolysate increased simultaneously with the incubation time. After Alcalase hydrolysis, the solubility of MPC reached the highest value (91.01%). The solubility of Fravourenzyme hydrolysates also increased significantly after hydrolysis, but the rate of increase was less than that of Alcalase and Protamex. Untreated MPC had the lowest solubility (48.23%). Generally, the conformational modification of protein molecules during storage is linked with the loss of solubility [34]. The reason for the solubility changes of MPC after enzymatic hydrolysis may be due to the caseins in MPC that are more sensitive to the enzymes compared with whey protein and are easily hydrolyzed by enzymes. In addition, interactions between the proteins may be reduced after hydrolysis, resulting in changes to the original characteristics of the MPC so that it increases in solubility.

### 3.2. SDS-PAGE

**Figure 1:**
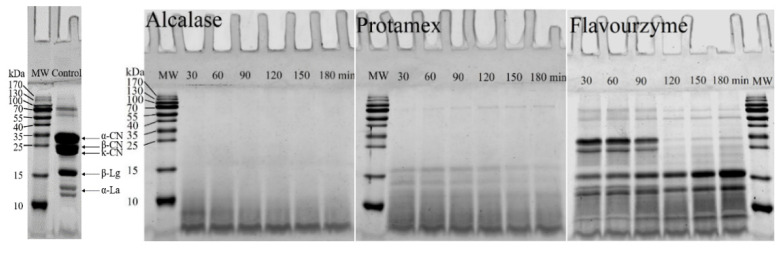
Sodium dodecyl sulfate–polyacrylamide gel electrophoresis (SDS-PAGE) of MPC and samples hydrolyzed by Alcalase, Flavorzyme, and Protamex at 30, 60, 90, 120, 150, and 180 min, respectively. The molecular weight of standard markers employed were 170, 130, 100, 70, 55, 40, 35, 25, 15, and 10 kDa, respectively.

The polypeptide profile of MPC hydrolysates after different enzymatic hydrolysis was analyzed by SDS-PAGE, and untreated MPC was included as the reference (Figure 1). As described in Figure 1, compared with the reference, the caseins were missing in the hydrolysates with Alcalase and Protamex because casein did not have a dense secondary and tertiary structure compared with whey protein. Alcalase hydrolyzed all the original proteins in MPC, including the compact whey proteins, to peptides with a molecular weight of less than 10 kDa. Zheng et al. [19] also observed that Alcalase was the most efficient in hydrolyzing the protein. Hydrolysis is easier under the action of endopeptidase [16]. Protamex has both endo- and exo-protease activity. The casein was also missing after hydrolysis with this enzyme, and whey proteins began to disappear gradually after 150 min of hydrolysis. Most peptides were less than 15 kDa. Flavourenzyme hydrolysates had the lowest hydrolysis efficiency, which might be because they lacked a high endopeptidase activity for proteolysis; instead, they had high exopeptidase activity [28]. Hydrolysis started from the end of the peptide, and the molecular weight did not change significantly. After 120 min of hydrolysis, peptides with a molecular weight of less than 15 kDa increased. Moreover, a new band was formed at less than 10 kDa for the hydrolysates with Flavourzyme.

In this study, endoproteases and proteases with both endo- and exoproteases were used. The endoproteases cut the peptide bond within the protein molecule, while the exoproteases broke the peptide bond of the terminal amino acid [33]. Alcalase has a high specificity for aromatic (Phe, Trp, and Tyr), acidic (Glu), sulfur-containing (Met), aliphatic (Leu and Ala), hydroxyl (Ser), and basic (Lys) residues, which preferentially hydrolyse peptide bonds containing aromatic amino acid residues [35]. Promatex contains very broad-spectrum endo-proteases, allowing extensive hydrolysis of proteins. Flavourzyme is produced from Aspergillus oryzae strains and is a mixture of endopeptidase and exopeptidase [36]. Therefore, MPC produced different hydrolysates following hydrolysis by various proteases. This is the main reason for the distinct structures and functional properties of the hydrolysates.

### 3.3. Surface Hydrophobicity

**Figure 2:**
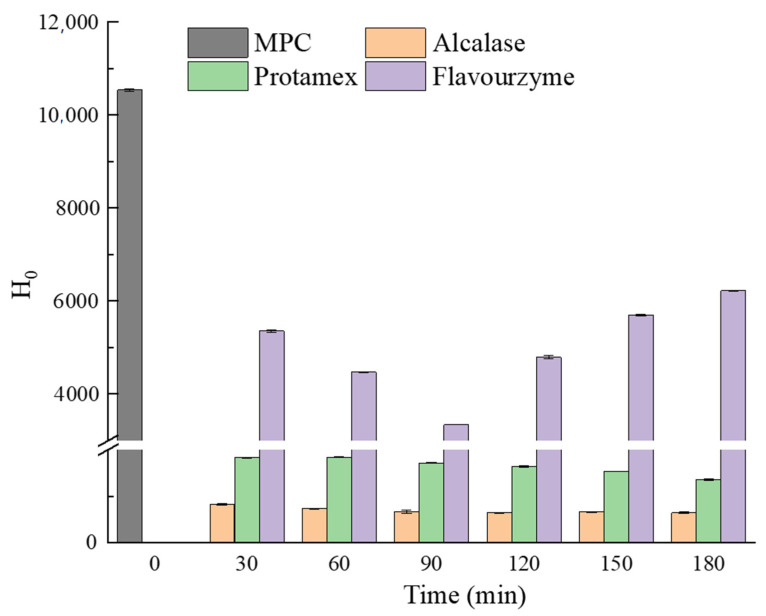
The surface hydrophobicity of control MPC and samples hydrolyzed by Flavorzyme, Alcalase, and Protamex at 30, 60, 90, 120, 150, and 180 min, respectively.

Surface hydrophobicity (H_0_) is one of the important influencing factors for protein structural properties, functional properties, and stability. The H_0_ of unmodified MPC and MPC hydrolysates is represented in Figure 2. The H_0_ of Alcalase hydrolysate was found to be the lowest. Alcalase preferentially hydrolyzes aromatic amino acid residues. The H_0_ of the Alcalase hydrolysate product was not protruding, which was related to the content of the anionic protein. The hydrolysis solution obtained by Flavourzyme had the highest H_0_. Compared with other enzymes, Flavourzyme could easily expose anion sites; therefore, the hydrolysate of Flavourzyme showed strong hydrophobicity [31]. After MPC was hydrolyzed by Alcalase and Protamex, H_0_ decreased with time. Zang, Yue, Wang, Shao, and Yu [37] found that H_0_ decreased with the increase in DH, which might be due to the free hydrophobic amino acids produced by enzymatic hydrolysis and the increased hydrophobic interaction leading to protein rearrangement. A change in surface hydrophobicity indicated the modified protein conformation. H_0_ increased after being hydrolyzed for 90 min by Flavourenzyme, which may be because of the exposed hydrophobic sites buried in the protein structure due to enzymatic cleavage [38]. Banach et al. [4] found that the decrease in the surface hydrophobicity contributed to the increased solubility. This was consistent with the results of the experiment. Proteins with fewer hydrophobic functional groups on the surface had better solubility in water. The change in surface hydrophobicity after enzymatic hydrolysis could be due to the release of more polar hydrophilic functional groups, thus improving the protein solubility [4].

### 3.4. Particle Size Distribution

The average particle size of the MPC hydrolysate is shown in Table 2. By testing the particle size, the dispersion or aggregation state of the MPC hydrolysate can be inferred. The particle size in the control group was 34.87 μm (unhydrolyzed). The average particle size of MPC decreased significantly after enzymatic hydrolysis (*p* < 0.05). The particle size value of Flavourzyme hydrolysate decreased with the extension of the hydrolysis time, and was the smallest after 180 min of hydrolysis. Sun et al. [15] found that the particle size of the untreated reconstituted MPC was 28.45 μm; this study was made on the freshly produced MPC, without storage. This value was lower than the MPC particle size value measured in our study, which might be because the particle size value of the MPC used in our work increased after long-term storage (6 months). Anema et al. [9] speculated that the insolubility of MPC may be caused by the protein cross-linking on the surface. MPC gradually decomposed during the enzymatic treatment, destroying the structure of the protein, and small peptides were more easily dissolved in the solution, increasing the solubility. The peptide further decomposed or aggregated with the extension of the hydrolysis time. Shen et al. [18] found that the particle size of soy protein increased with the increase in DH after Alcalase hydrolysis. Similarly, the particle size of MPC increased after hydrolysis with Alcalase and Protamex.

### 3.5. Relative Fluorescence Intensity and Ultraviolet Absorption Spectroscopy

**Figure 3:**
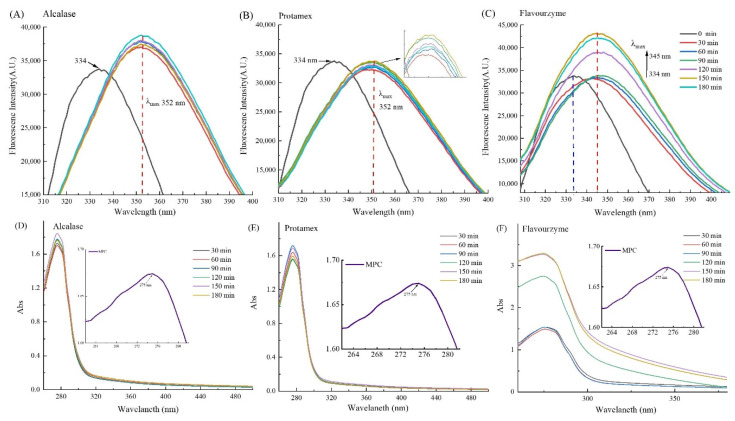
Structural characterization of MPC and hydrolysates. Relative fluorescence spectra of milk protein concentrate and hydrolysates prepared with Alcalase (**A**), Protamex (**B**), and Flavorzyme (**C**); as well as UV–visible spectra of MPC and hydrolysates prepared with Alcalase (**D**), Protamex (**E**), and Flavorzyme (**F**).

Intrinsic fluorescence of protein is influenced by the polarity of the chromophores in the environment, which is related to the exposure of aromatic amino acids to water [39]. Figure 3A–C) shows the intrinsic fluorescence spectra of untreated MPC and different enzymatic hydrolysates. The peak value of the enzymatic hydrolysis solution exhibited a redshift compared with the untreated MPC (334 nm) fluorescence peak, which indicated that the conformation of the protein system after enzymatic hydrolysis changed, and the fluorophore shifted to a more hydrophobic environment [39]. Compared with Flavourzyme, the peak of the fluorescence spectrum after Alcalase and Protamex treatment had a greater degree of redshift, which was consistent with the results of H_0_. Moreover, the MPC hydrolysates obtained from Flavourzyme digestion presented much higher fluorescence emission spectra compared with the emission spectra of Alcalase- and Protamex-treated hydrolysates. The Flavourzyme-treated protein hydrolysates exposed the anion site flexibility. The relative fluorescence intensity might be related to the specificity of each enzyme [17].

The results of ultraviolet (UV) absorption spectra of MPC hydrolysates obtained with Alcalase, Protamex, and Flavourzyme are shown in Figure 3D–F). The maximum absorption peak of untreated MPC was at 275 nm. Predictably, the enzymatic hydrolysis caused a significant shift in the UV absorption bands. This shift was most likely caused by the conformational changes in protein after enzymatic treatment [19]. Compared with Alcalase and Protamex, the UV spectrum of Flavourzyme hydrolysis was significantly different, which highlighted that the hydrolyzed products of MPC were different for different enzymes.

### 3.6. FTIR

**Figure 4:**
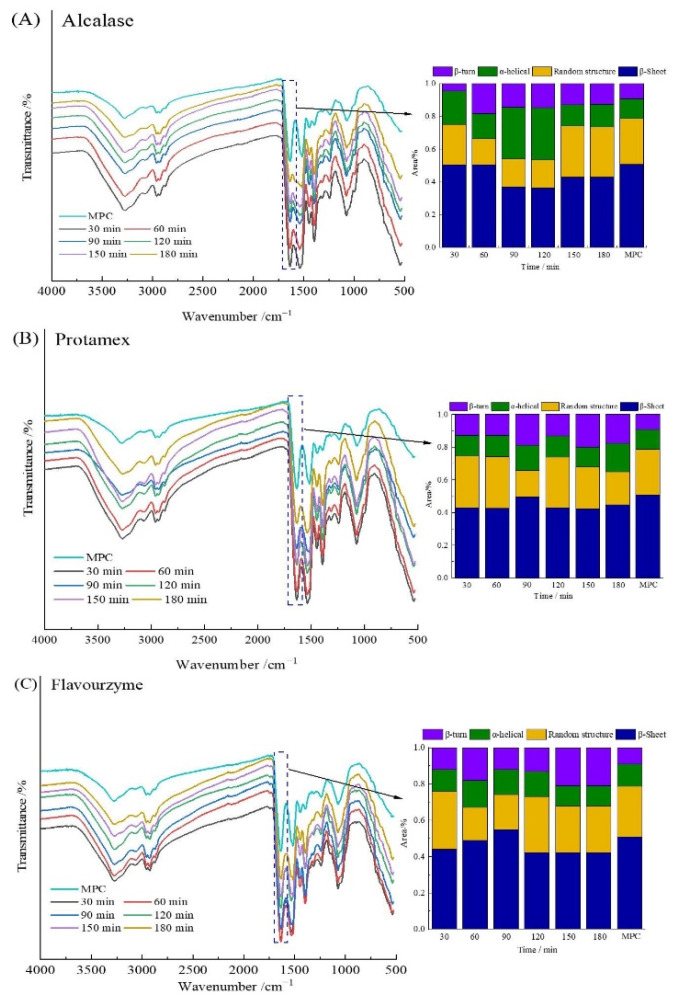
Structural characterization of MPC and hydrolysates. FTIR spectra and relative content of the secondary structure of MPC and hydrolysates prepared with: Alcalase (**A**); Protamex (**B**); and Flavorzyme (**C**).

Figure 4 reflects the FTIR spectra of different hydrolysates. The FTIR spectrum could display protein amide I band, amide II band, and amide III band information, as well as C-C stretching vibration and C = O in the protein structure. The main spectra associated with the peptides produced with Alcalase, Protamex, and Flavourzyme were 3269–3280 cm^−1^ (N-H stretch), 2928 cm^−1^ (O-H stretch), and 1633 cm^−1^ (C = O stretch) related to the amide region I; 1515–1535 cm^−1^ related to the N-H deformation and C-N stretch at amide II vibrations; and 1070–1075 cm^−1^ related to the C-O stretch, respectively. The different hydrolysates of MPC all peaked at 3200–3300 cm^−1^ [27,40]. The band of amide I in the infrared spectrum was the main band of protein secondary structure in the range of 1600–1700 cm^−1^.

The amide I region was divided into β-sheet (1618–1640 cm^−1^ and 1670–1690 cm^−1^), random structure (1640–1650 cm^−1^), α-helical (1650–1660 cm^−1^), and β-turn (1660–1670 cm^−1^ and 1690–1700 cm^−1^) structures [31,41]. Qi et al. [40] showed that the secondary structure of untreated MPC was β-sheet (51%), random structure (15%), α- helical (15%), and β-turn (20%). However, the secondary structure of each hydrolysate did not remain the same after hydrolysis by various proteases. The content of β-sheet in the hydrolysate was the highest after hydrolysis by the three enzymes, and the content of α-helical also increased slightly. The difference in the hydrolysis site and DH of different enzymes was the main reason for the variation in the secondary structure composition after enzymatic hydrolysis.

### 3.7. Representation of Sensor Response of the Electronic Tongue

The electronic tongue system has unique capabilities; the most important of these is the ability to deal with the complex and changing background and reduce the influence of interference [42]. According to the output from the electronic tongue in Table 3, the hydrolysis with proteases increased the bitterness of the hydrolysates in relation to control. Alcalase had the highest bitterness value, while Protamex had a relatively lower bitterness value. The bitterness values of the protein hydrolysates with Alcalase were maintained at similar levels between 30 to 180 min of hydrolysis. However, no apparent relationship existed between the molecular size of the peptides and bitterness [43]. The bitterness might be related to the specificity of each enzyme. Alcalase had a broad specificity for peptides formed by hydrophobic amino acids, the hydrolysates of which saw an increase in protein flexibility and aromatic site exposure. This may be the reason for the bitterness of Alcalase hydrolysate in the study. The sensor output of the bitter astringency, aftertaste-B and astringent aftertaste-A, correlated with the hydrophobicity of bitter peptides. The bitterness characterization (aftertaste-B) of Protamex hydrolysate was of a significantly lower level than that of hydrolysates produced by Alcalase and Flavourzyme, respectively. The release of free amino acids by Flavourzyme could also have contributed to the high level of bitterness within its hydrolysate. Thus, Protamex was the enzyme that produced hydrolysates with the lowest levels of bitterness.

### 3.8. Correlation Analysis

**Figure 5:**
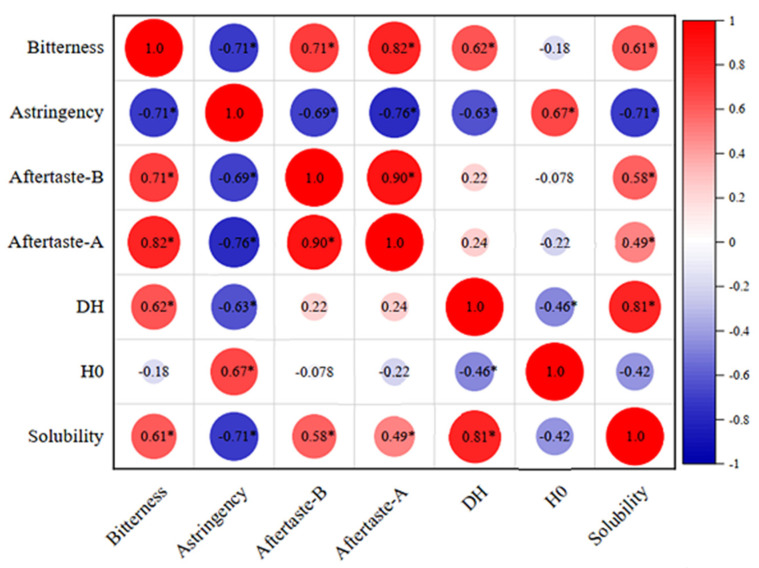
Heatmap summarizing correlation coefficients between different indicators. Red represents a positive correlation, blue represents a negative correlation, and the depth of color reflects the magnitude of the correlation coefficient. Pearson’s correlation analyses were performed. The R values are shown in different colors in the figure, and the right legend provides a color interval with different R values. The *p* values are marked with asterisks (*) for *p* < 0.05.

The relationship between bitterness and different indicators of MPC hydrolysates was affected by many factors, such as enzyme activity, hydrolysis time, and enzyme type. As shown in Figure 5, a positive correlation (r = 0.62) existed between bitterness and DH, which was in accordance with a previous study [44]. Proteins were hydrolyzed by proteases into small peptides, and the main substances producing bitterness were short peptides. Analysis of the correlations between the sensor outputs by the electronic tongue (Figure 5) showed a significant positive association (r = 0.71 and r = 0.82) between bitterness and aftertaste (aftertaste-B) and astringent aftertaste (aftertaste-A). The aftertaste (aftertaste-B) and astringent aftertaste (aftertaste-A) reflected the bitterness, which was related to the specificity of each enzyme. According to the analysis by electronic tongue in Table 3, the Alcalase hydrolysate had the highest bitterness. Protein solubility had a positive correlation (r = 0.81 and r = 0.61) with DH and bitterness. Interactions between protein molecules may be broken after hydrolysis, increasing the DH and protein solubility, but also increasing the bitterness value.

## 4. Conclusions

The results indicated that in the hydrolysates of MPC obtained by treatment with Alcalase, the endopeptidase presented higher DH than those obtained by Protamex and Flavourzyme. The protein solubility of all hydrolysates significantly increased. In addition to solubility, the bitterness of the hydrolysate is another main factor affecting the practical application of MPC. The solubility of hydrolysates of MPC was strongly correlated with DH and bitterness, especially solubility and DH. Compared with Alcalase and Flavourenzyme, the treatment with Protamex led to the hydrolysates with the lowest levels of bitterness. Considering solubility and bitterness properties, Protamex incubation for 120 min is best to hydrolyze MPC for use as an ingredient in different products. These results may help to better understand the effect of enzymes on the bitterness and solubility of MPC hydrolysates. This work could provide a new perspective on MPC utilization and the potential application of MPC hydrolysates as a protein ingredient for food formulations.

## Figures and Tables

**Table 1:** Degree of hydrolysis and solubility of should be milk protein concentrate (MPC) hydrolysates by Alcalase, Flavourzyme, and Protamex.

Sample (min)	DH			Solubility		
	Alcalase	Protamex	Flavourzyme	Alcalase	Protamex	Flavourzyme
control	-	-	-	48.2 ± 0.51a	48.2 ± 0.51a	48.2 ± 0.51a
30	12.9 ± 0.51a	10.1 ± 0.97a	9.9 ± 0.79a	72.1 ± 2.3b	68.2 ± 1.1d	64.3 ± 0.19b
60	13.2 ± 0.42a	12.60 ± 1.2ab	10.1 ± 1.1a	74.1 ± 1.1c	61.6 ± 2.1b	64.9 ± 1.2b
90	14.7 ± 1.1ab	13.3 ± 0.76b	10.4 ± 1.1a	78.0 ± 3.20d	67.3 ± 1.2d	67.6 ± 0.87c
120	15.3 ± 0.95b	13.3 ± 0.83b	11.5 ± 1.1a	86.3 ± 2.1e	64.1 ± 2.2c	70.8 ± 0.94d
150	15.6 ± 0.12b	13.4 ± 1.2b	11.6 ± 0.21a	90.2 ± 1.1f	81.1 ± 1.1e	72.6 ± 1.2d
180	15.7 ± 1.3b	13.6 ± 0.93b	11.9 ± 0.13a	91.1 ± 0.91f	85.1 ± 1.2f	75.2 ± 2.1e

All experiments were done in parallel for at least three times. Data represent mean ± standard deviations. Different lowercase letters in the same column indicate the significant differences of different hydrolysis times (*p* < 0.05).

**Table 2:** Particle size of MPC hydrolysates by Alcalase, Flavorzyme, and Protamex.

Sample (min)	Particle Size (nm)		
	Alcalase	Protamex	Flavourzyme
control	34.87 ± 1.21 (μm)		
30	245.8 ± 2.9a	226.4 ± 4.6bc	220.1 ± 4.3c
60	261.7 ± 1.5b	221.3 ± 1.7ab	213.3 ± 4.9c
90	258.8 ± 41b	218.9 ± 3.9a	221.7 ± 16.8c
120	261.9 ± 4.20b	229.1 ± 4.3cd	221.1 ± 3.4c
150	275.8 ± 3.2c	243.9 ± 2.6e	199.8 ± 1.1b
180	286.9 ± 3.3d	234.7 ± 3.3d	183.3 ± 2.3a

All experiments were done in parallel for at least three times. Data represent mean ± standard deviations. Different lowercase letters in the same column indicate the significant differences between various hydrolysis times (*p* < 0.05).

**Table 3:** The sensor outputs of MPC hydrolysates obtained by the electronic tongue.

Sample (min)	Alcalase				Protamex				Flavourzyme			
	Bitterness	Aftertaste−B	Astringency	Aftertaste−A	Bitterness	Aftertaste−B	Astringency	Aftertaste−A	Bitterness	Aftertaste−B	Astringency	Aftertaste−A
control	4.88 ± 0.46a	2.76 ± 0.87a	−3.29 ± 0.06c	−1.65 ± 0.25a	4.88 ± 0.46a	2.76 ± 0.87a	−3.29 ± 0.06d	−1.65 ± 0.25a	4.88 ± 0.46a	2.76 ± 0.87ab	−3.29 ± 0.06b	−1.65 ± 0.25a
30	8.22 ± 0.05b	3.8 ± 0.06ab	−6.81 ± 0.03b	−1.14 ± 0.17ab	6.48 ± 0.5b	1.81 ± 1.2a	−3.88 ± 0.09c	−2.08 ± 1.18a	7.71 ± 0.08c	2.43 ± 0.17a	−2.96 ± 0.09b	−1.5 ± 0.11a
60	8.40 ± 0.04b	4.16 ± 0.18ab	−6.8 ± 0.05b	−1.06 ± 0.18ab	6.42 ± 0.51b	1.61 ± 1.3a	−4.01 ± 0.11bc	−2.1 ± 1.3a	7.57 ± 0.03ab	2.59 ± 0.17ab	−3.47 ± 0.16a	−1.55 ± 0.16a
90	8.25 ± 0.08b	3.71 ± 0.12ab	−6.87 ± 0.09b	−1.03 ± 0.31bab	6.32 ± 0.68b	1.04 ± 0.12a	−4.13 ± 0.12abc	−2.17 ± 1.4a	7.47 ± 0.28ab	2.86 ± 0.37ab	−3.67 ± 0.25a	−1.64 ± 0.40a
120	8.14 ± 0.12b	3.96 ± 0.64ab	−6.92 ± 0.14b	−1.07 ± 0.64ab	6.18 ± 0.74b	0.99 ± 0.12a	−4.19 ± 0.12abc	−2.29 ± 1.7a	7.18 ± 0.14b	3.06 ± 0.12ab	−4.15 ± 0.11a	−1.68 ± 0.24a
150	8.41 ± 0.29b	4.30 ± 0.75b	−6.91 ± 0.04b	−0.87 ± 0.75b	6.36 ± 0.12b	2.63 ± 0.21a	−4.32 ± 0.04ab	−1.99 ± 0.04a	7.39 ± 0.12ab	3.95 ± 0.30c	−4.24 ± 0.21a	−1.45 ± 0.43a
180	8.11 ± 0.38b	3.95 ± 0.96ab	−7.09 ± 0.07a	−0.99 ± 0.96b	6.50 ± 0.04b	2.78 ± 0.34a	−4.41 ± 0.08a	−2.01 ± 0.10a	7.14 ± 0.36b	3.49 ± 0.58bc	−4.46 ± 0.25a	−1.68 ± 0.50a

All experiments were done in parallel for at least three times. Data represent mean ± standard deviations. Different lowercase letters in the same column indicate the significant differences of different hydrolysis times (*p* < 0.05).
